# Sporadic Lymphangioleiomyomatosis in a 35-Year-Old Female Patient: A Rare Cause of Spontaneous Pneumothorax

**DOI:** 10.7759/cureus.87346

**Published:** 2025-07-05

**Authors:** Peter N Rodenko, Josh Elefteratos, Emily L Rodenko, Colton Herrell, Timothy Townsend

**Affiliations:** 1 Medicine, St. George's University School of Medicine, St. George, GRD; 2 Internal Medicine, St. George's University School of Medicine, St. George, GRD; 3 Biology, Trinity University, San Antonio, USA; 4 Radiology, Medical Center Health System, Odessa, USA

**Keywords:** cystic lung disease, lam, lam pneumothorax, lymphangioleiomyomatosis, lymphangioleiomyomatosis pneumothorax, s-lam, sporadic lam, sporadic lymphangioleiomyomatosis

## Abstract

Lymphangioleiomyomatosis (LAM) is a rare, progressive cystic lung disease that primarily affects women of reproductive age. We report a case of a 35-year-old Caucasian female with a history of seasonal nasal allergies who presented to the emergency department with acute-onset shortness of breath and a chronic dry cough. The acute episode closely resembled a prior hospitalization one month earlier for spontaneous pneumothorax, which had been managed with a pigtail catheter. During this admission, physical exam revealed absent breath sounds in the right upper lung fields and base. Chest X-ray confirmed a recurrence of her right-sided pneumothorax, and a chest tube was inserted after unsuccessful pigtail catheter placement. Contrast-enhanced chest CT showed multiple, discrete, thin-walled cystic lesions diffusely distributed throughout the lungs, supporting a diagnosis of LAM over emphysema. Mechanical pleurodesis and thoracoscopic biopsy were performed, with pathology confirming LAM.

LAM is frequently underdiagnosed due to its low incidence and often nonspecific symptoms. Pneumothorax can be a common early manifestation and is often the first clinical clue, as in this case. High-resolution CT imaging typically shows homogeneous, thin-walled cysts throughout the lungs. Contrast-enhanced CT displayed similar findings in this case. Sirolimus, a mammalian target of rapamycin (mTOR) inhibitor, was initiated in the outpatient setting, and it is the standard of care for stabilizing lung function and reducing disease progression in patients afflicted by LAM. This report emphasizes the importance of considering LAM in young women presenting with spontaneous pneumothorax. Early detection of LAM may lead to more favorable outcomes, with management focused on preserving lung function, preventing complications, and educating patients, as emerging molecular therapies further refine treatment strategies.

## Introduction

Lymphangioleiomyomatosis (LAM) is a rare, systemic, and progressive neoplastic disorder characterized by the abnormal proliferation of spindle-shaped smooth muscle-like cells. These cells infiltrate the lungs, kidneys, and lymphatic system, resulting in diffuse thin-walled pulmonary cysts, renal angiomyolipomas, and various lymphatic abnormalities. The disease has a pronounced female gender bias, predominantly affecting women of reproductive age; LAM in males is exceedingly uncommon, with only 36 male cases documented in the literature since 1986 [1]. In recent years, even though the detection and understanding of LAM have increased, this disease remains rare, with fewer than seven cases per million individuals reported in the general population [2]. LAM is associated with significant morbidity and chronicity. The transplant-free median survival is approximately 29 years, and the 10-year survival rate is reported to be 86% [3].

LAM cells infiltrate the pulmonary parenchyma, vasculature, and airways, contributing to vascular remodeling and cyst formation. These cysts are typically discrete, round, and thin-walled, which predisposes them to rupture, often leading to clinical presentations of recurrent spontaneous pneumothorax and progressive dyspnea [3]. Extrapulmonary manifestations include renal angiomyolipomas, which are more frequent in tuberous sclerosis-associated LAM (TSC-LAM) [4,5]. Lymphatic complications include chylothorax due to lymphatic congestion and lymphangioleiomyomas, which most commonly form in the retroperitoneum, pelvis, or posterior mediastinum [5].

LAM has been associated with TSC1 and TSC2 gene mutations, which encode the tumor suppressor proteins hamartin and tuberin [6]. These mutations lead to dysregulation of the mammalian target of the rapamycin (mTOR) signaling pathway, resulting in unchecked cellular growth and proliferation [5,6]. Two distinct forms of LAM have been described: TSC-LAM and sporadic LAM (S-LAM) [7]. TSC-LAM occurs in the setting of tuberous sclerosis complex (TSC), a multisystem genetic disorder characterized by mutations in TSC1 and TSC2, and is associated with additional clinical features including shagreen patches, facial angiofibromas, and recurrent seizures. In contrast, S-LAM arises from somatic TSC2 mutations and lacks the multisystem involvement typical of TSC [5].

The characteristic cells produced in LAM (termed LAM cells) exhibit dual differentiation, with desmin-positive smooth muscle features internally and HMB-45-positive melanocytic markers externally; additionally, LAM lung nodules and LAM-associated angiomyolipomas have shown a propensity to be estrogen-sensitive [8,9]. While the only known curative treatment for LAM is lung transplantation, mTOR pathway inhibitors like sirolimus are the mainstay of management for patients with LAM, along with supportive therapy such as bronchodilators, supplemental oxygen, and pulmonary rehabilitation [10]. This report illustrates the classical presentation and clinical course of this pathology in a female of childbearing age.

## Case presentation

This patient was a 35-year-old Caucasian female with a past medical history of nasal allergies who presented to the emergency room with shortness of breath and dry cough, which had been ongoing for two months. The shortness of breath had started acutely while at rest after a coughing episode and worsened with exertion. She did not report any wheezing, chills, fever, chest pain, palpitations, edema, headache, or recent trauma. The patient had been hospitalized one month before this admission due to shortness of breath. During this previous admission, a spontaneous pneumothorax had been diagnosed and treated with a pigtail catheter, leading to symptomatic relief and same-day discharge. On this new admission, the patient reported that her current symptoms resembled those experienced during her prior hospitalization, which had prompted her to return to the emergency room. 

The patient had no significant past medical history aside from seasonal nasal allergies for which she was taking cetirizine as needed and fluticasone. The patient was taking no other medications and had no known allergies to food or medication. There was no significant family history of disease or cancer. Surgical history included a C-section, tonsillectomy, adenoidectomy in childhood, and a thoracostomy tube placed one month prior for spontaneous pneumothorax. The patient had never used alcohol, tobacco, or illicit drugs.

**Figure 1:**
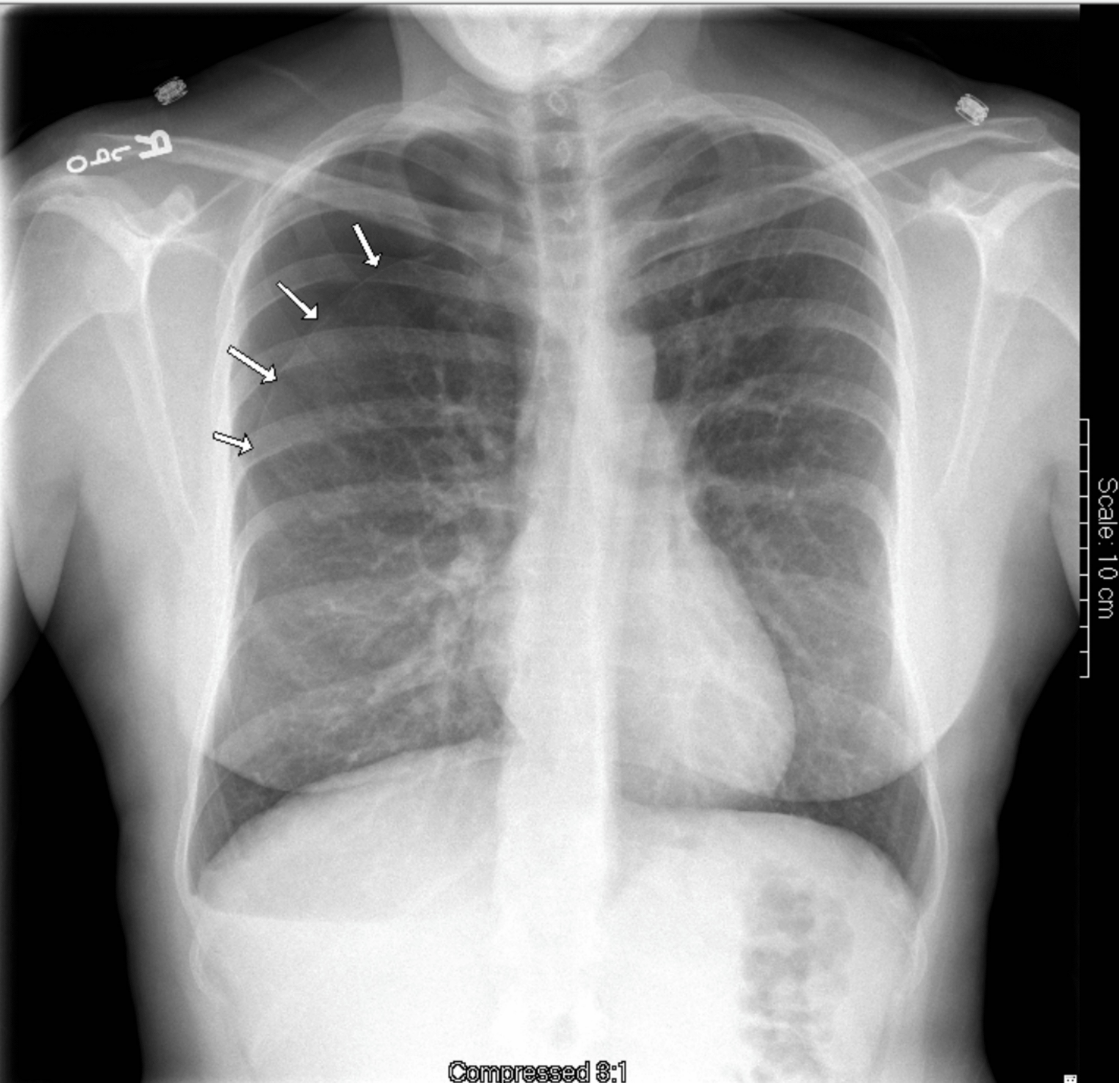
PA chest X-ray on initial presentation There is an appreciable moderate-sized pneumothorax in the right upper lung, as shown by the single arrows PA: posteroanterior

**Figure 2:**
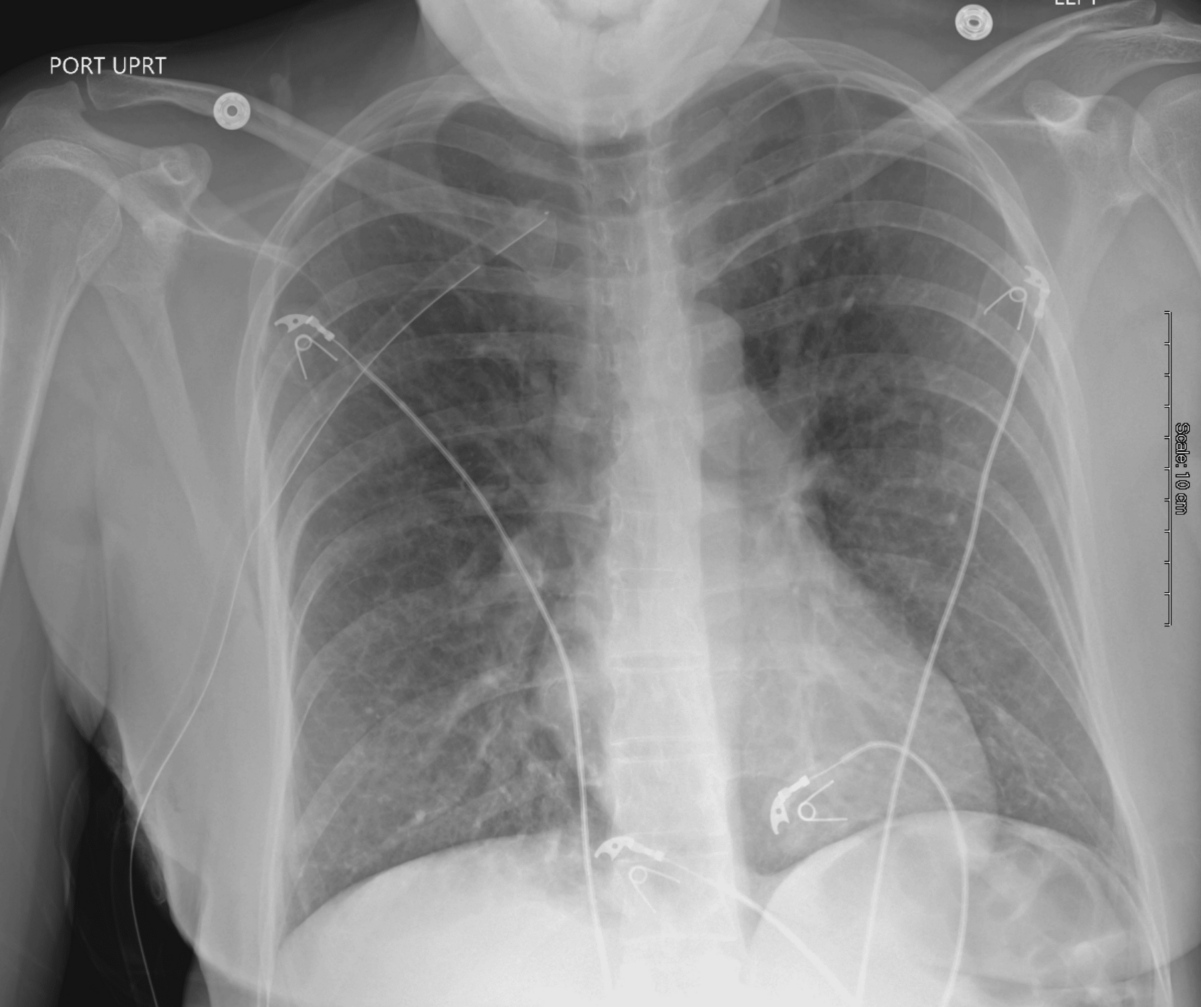
AP chest X-ray after chest tube insertion showing resolution of pneumothorax with adequate chest tube placement AP: anteroposterior

During examination, the patient was alert and oriented, with labored breathing but otherwise in no acute distress. Vital signs and temperature were within normal limits, including an oxygen saturation of 98% and a respiratory rate of 18 breaths per minute. Physical exam yielded normal cardiac, airway, abdominal, musculoskeletal, neurological, and peripheral vasculature findings. There was a subtle increase in the work of breathing. Upon lung auscultation, there was an absence of breath sounds in the upper right lung fields and diminished lung sounds in the right lung base. Labs on admission were unremarkable. A chest X-ray (Figure 1) showed a right upper lung pneumothorax. A pigtail catheter was first attempted for thoracostomy but was unsuccessful, prompting chest tube insertion, which was successful. The post-chest tube placement X-ray is shown in Figure 2.

**Figure 3:**
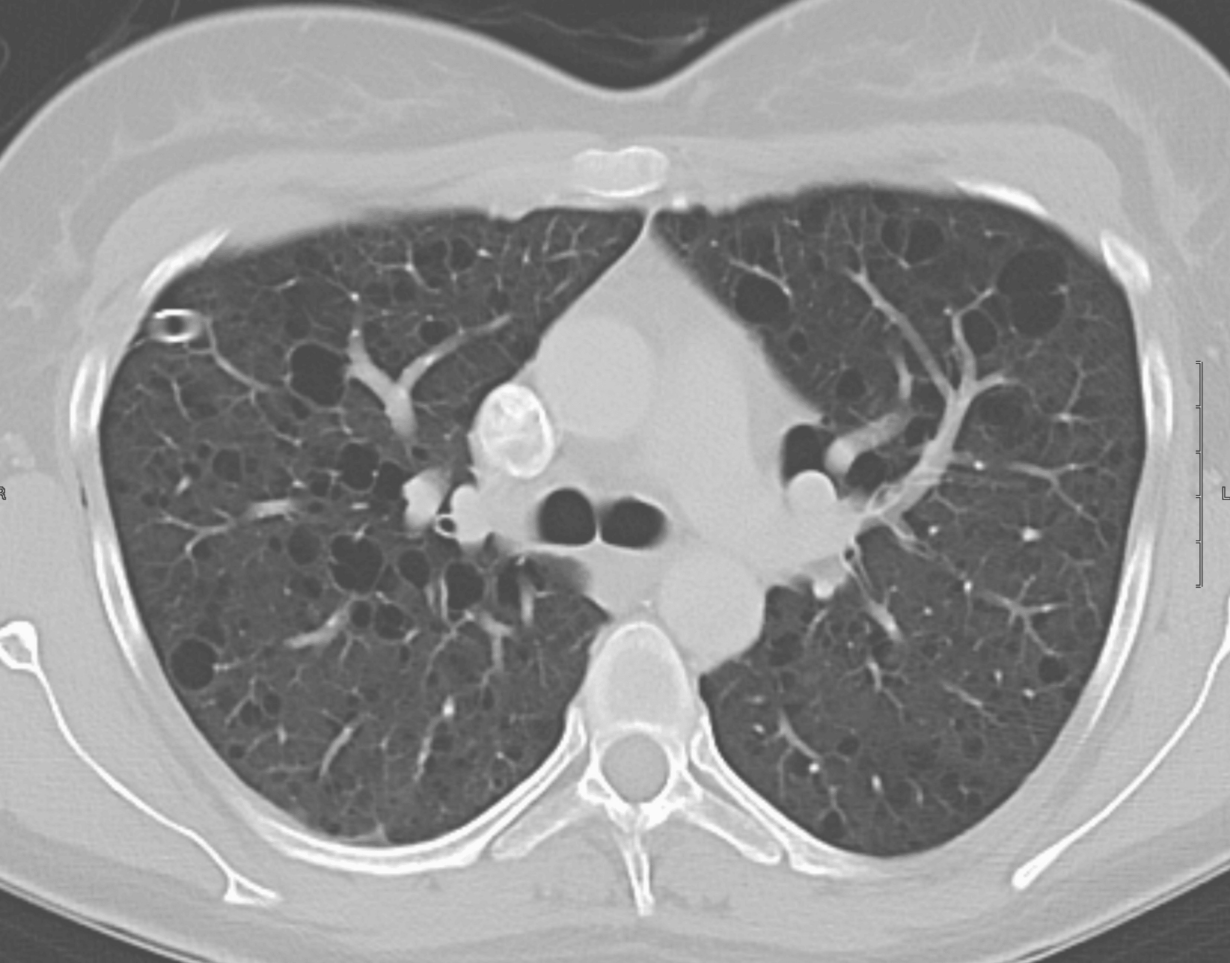
Contrast-enhanced CT of the upper lung zones showing numerous bilateral discrete cystic spaces with very thin or imperceptible walls Lung volumes are normal with no evidence of emphysema CT: computed tomography

**Figure 4:**
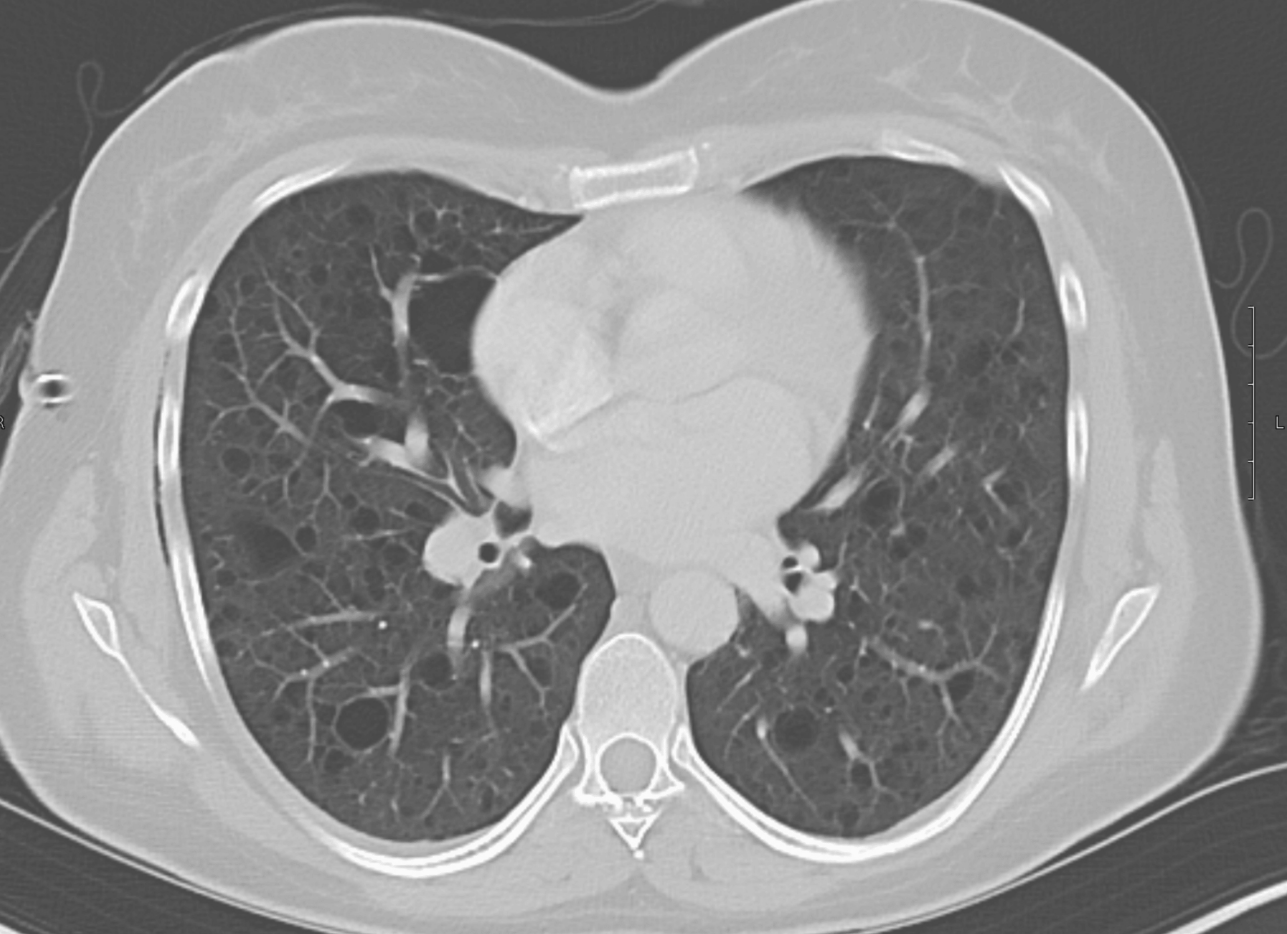
Contrast-enhanced CT of the middle lung zones showing numerous bilateral discrete cystic spaces with very thin or imperceptible walls Lung volumes are normal with no evidence of emphysema CT: computed tomography

**Figure 5:**
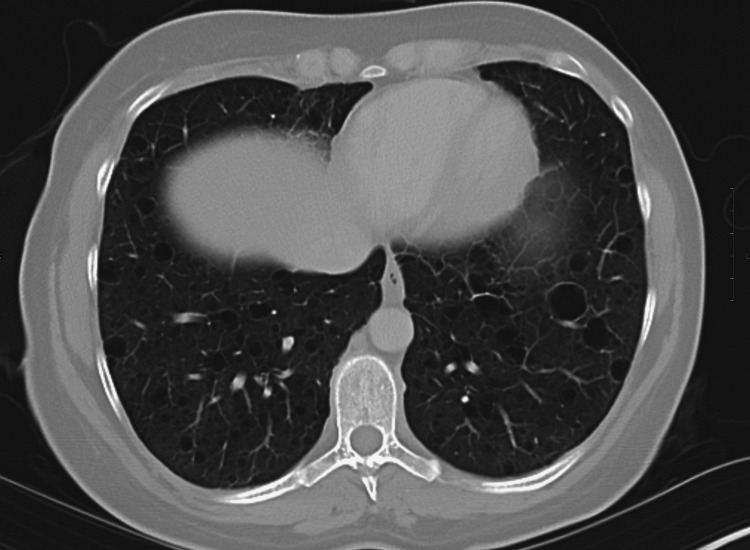
Contrast-enhanced CT of the lower lung zones showing numerous bilateral discrete cystic spaces with very thin or imperceptible walls Lung volumes are normal with no evidence of emphysema. CT: computed tomography

Due to the patient’s history of prior pneumothorax, further workup was needed to determine the cause of recurrent spontaneous pneumothorax in a young non-smoking female with no significant medical history. A contrast-enhanced chest CT was ordered (Figures 3-5), and surgery to prevent recurrence was planned. Chest CT showed multiple discrete cystic spaces with thin or imperceptible walls throughout both lungs in the upper, middle, and lower lung zones. These cystic structures looked similar to bullae but lacked marginal irregularities, indicating they were most likely thin-walled cysts. These structures had no predominance in the upper or lower lung zones. Chest CT was otherwise unremarkable. Emphysematous changes were unlikely as there was no lung hyperinflation, distortion of pulmonary vessels, or right ventricular enlargement.

The CT findings supported the diagnosis of LAM, and further imaging with abdominal and pelvic CT was unremarkable for any renal angiomyolipomas or other findings classically associated with tuberous sclerosis or LAM. Pleurodesis was indicated to prevent pneumothorax recurrence, and concurrent biopsy was suggested to further support the diagnosis. Mechanical pleurodesis was favored over talc pleurodesis due to the latter potentially complicating future thoracic surgeries. Thoracoscopic right upper lung biopsy and mechanical pleurodesis were performed successfully. During the surgery, diffuse cystic disease of all lung lobes was noted, with no pleural fluid and minimal adhesions within the thoracic cavity. 

Histopathology report of wedge biopsy sections collected from the right upper lung showed dilated alveolar spaces with few cystic areas and multinodular proliferations of bland spindle cells. Additionally, the samples showed focal and weak estrogen receptor (ER) sensitivity and strong positive staining with desmin, podoplanin, and HMB-45. Melan-A and S100 markers were not identified on staining. The combined morphologic and histologic features raised a strong suspicion for early-stage LAM. The patient denied genetic testing for tuberous sclerosis, but sporadic LAM was the primary differential, given that the patient lacked a family history of tuberous sclerosis or any other symptoms/findings associated with it. The patient followed up with an outpatient pulmonologist and oncologist who prescribed sirolimus for long-term management with continued monitoring.

## Discussion

We discussed a case of a young adult female with a confirmed diagnosis of LAM, a rare and progressive cystic lung disease that primarily affects women of reproductive age. The rarity of this diagnosis is well-supported by epidemiological data, with studies across seven countries estimating a prevalence of only three to seven cases per one million people [2]. Furthermore, the sporadic form of LAM has been reported to occur in approximately one in 400,000 women [11]. Given these exceedingly low prevalence rates, the manifestation of sporadic LAM in this patient highlights the clinical rarity and diagnostic challenge associated with this condition. In this patient, the initial chest X-ray displayed mildly increased interstitial markings throughout both lung fields with preservation of lung volumes and a moderately sized right lung pneumothorax (Figure 1). Chest X-ray findings in LAM are often nonspecific, and underlying cystic lung changes are more easily visualized on CT. Contrast-enhanced CT of the chest revealed multiple, well-defined, thin-walled cystic lesions that were diffusely distributed throughout both lungs. These findings are characteristic of LAM and support the clinical diagnosis in the context of the patient's initial presentation [9].

The absence of significant nodules, ground glass opacities, or consolidations supports a diagnosis of LAM as opposed to other cystic lung diseases, notably Langerhans cell histiocytosis or Birt-Hogg-Dubé syndrome [10]. While the imaging may superficially resemble emphysema, the presence of discrete, thin-walled cysts without zonal predominance differentiates LAM from more common causes of cystic lung disease [12]. LAM involves uniformly distributed cysts with preserved lung structure, which contrasts with emphysematous changes due to smoking and alpha-1 antitrypsin (A1AT) deficiency that present with areas of parenchymal destruction and have a predominance for the upper and lower lobes, respectively [12]. In a 35-year-old nonsmoking female, emphysema is an unlikely primary diagnosis, though A1AT deficiency should be considered given its potential to cause early-onset emphysema in nonsmokers [13,14]. 

LAM was confirmed through biopsy and histopathological analysis, which continues to be the gold standard for establishing a definitive diagnosis. While high-resolution CT is the imaging modality of choice in the assessment of LAM, contrast-enhanced CT was employed in this case without compromising the accuracy of the diagnosis. This patient initially presented with a spontaneous pneumothorax, which is among the most frequent complications of LAM [13]. Pneumothoraces occur in up to 70% of patients with LAM and may be the first clinical manifestation of the disease [15]. Recurrent spontaneous pneumothoraces typically result from the rupture of subpleural cysts, warranting prophylactic pleurodesis, as performed in this patient. Beyond pneumothorax, LAM is associated with other serious complications, including chylous effusions, renal angiomyolipomas, and respiratory failure [10]. These complications highlight LAM's systemic nature and reinforce the need for a multidisciplinary approach to diagnosis and management. The presence of a pneumothorax in this case not only supported the need for additional imaging but was consistent with the known course and predisposed demographic of LAM, corroborating clinical suspicion and subsequent diagnosis. 

The management of LAM focuses on slowing disease progression, preserving lung function, and addressing complications. Regular monitoring of lung function is essential, as LAM can progress to respiratory failure over time [10]. Genetic testing for mutations in the TSC1 and TSC2 genes is important, given that LAM has been associated with tuberous sclerosis. In this case, the patient was initiated on sirolimus, an mTOR inhibitor that is considered the standard of care for patients with advanced disease: those with impaired lung function, lymphatic involvement, or renal angiomyolipomas [4]. Sirolimus has been shown to stabilize lung function, reduce cystic lung damage, and shrink angiomyolipomas, ultimately improving quality of life in affected patients [16].

This patient was referred for ongoing outpatient follow-up, including pulmonary function testing every three to six months. Additional monitoring of VEGF-D levels and regular analysis of sirolimus levels, renal function, liver enzymes, and lipid profiles for drug-related adverse effects is recommended [16]. Supportive care also plays a critical role and may include bronchodilators, oxygen therapy when indicated, and adherence to vaccination guidelines for immunocompromised individuals [10]. A notable aspect of LAM management is the strict avoidance of estrogen exposure, particularly in the form of hormonal contraception and hormone replacement therapy, which is relevant in the reproductive age female demographic in whom LAM most commonly occurs [9]. This recommendation stems from the estrogen-sensitive nature of LAM. Lung transplantation may be considered in advanced cases with severe respiratory decompensation, and it remains the only curative measure for LAM [13]. 

While current management of LAM focuses on mTOR inhibition with sirolimus, future directions aim to explore alternative mTOR inhibitors, such as everolimus, which may offer comparable efficacy with a reduced side effect profile [10]. There is increasing interest in targeting upstream pathways involved in LAM pathogenesis, including VEGF-D signaling and the autophagy-lysosomal pathways [4]. Moreover, taking into account the estrogen-sensitive nature of LAMs, selective estrogen receptor modulators have been investigated, though current evidence remains inconclusive [9]. Looking forward, there is growing emphasis on individualized treatment plans based on genetic and molecular profiling, especially in patients with TSC-associated LAM [10]. As therapeutic advances improve life expectancy, attention is also shifting towards preserving quality of life, managing long-term effects of therapy, addressing fertility concerns, and patient education [10].

## Conclusions

This report highlights the complexity and diagnostic nuances of LAM, a rare and progressive disease with diverse pulmonary and extrapulmonary manifestations. This patient’s initial presentation with spontaneous pneumothorax, diffuse cystic lung changes on contrast-enhanced CT, and the absence of conventional risk factors for emphysema exemplifies the diagnostic difficulty LAM can pose in young, nonsmoking women. Effective management requires a comprehensive approach that integrates imaging, genetic testing, and pulmonary function assessments, with sirolimus representing the mainstay of treatment. Ongoing surveillance, supportive intervention, and lifestyle considerations such as estrogen avoidance are equally essential in optimizing patient outcomes and maintaining quality of life. As the understanding of LAM’s molecular underpinnings continues to develop, future therapies and precision medicine strategies are expected to enhance the scope and specificity of treatment. Ultimately, proactive clinical collaboration and management remain critical to caring for individuals affected by this uncommon yet significant condition.
